# Interlaminar Toughening of Epoxy Carbon Fiber Reinforced Laminates: Soluble Versus Non-Soluble Veils

**DOI:** 10.3390/polym11061029

**Published:** 2019-06-11

**Authors:** Giulia Ognibene, Alberta Latteri, Salvatore Mannino, Lorena Saitta, Giuseppe Recca, Fabrizio Scarpa, Gianluca Cicala

**Affiliations:** 1 Department of Civil Engineering and Architecture, University of Catania, V.le A. Doria 6, 95125 Catania, Italy; giuliaognibene@live.com (G.O.); alatteri@unict.it (A.L.); salvatoremannino@hotmail.com (S.M.); lorena.saitta@icloud.com (L.S.); 2 CNR-ICPB, Via Paolo Gaifami 17, 95125 Catania, Italy; giuseppe.recca@cnr.it; 3 Bristol Composites Institute (ACCIS), University of Bristol, University Walk, BS8 1TR Bristol, UK; F.Scarpa@bristol.ac.ul

**Keywords:** electrospinning, epoxy, composites, interlaminar toughening

## Abstract

This work describes the evaluation of different interlaminar veils to improve the toughening of epoxy/carbon fiber composites manufactured by resin infusion. Three commercial veils have been used in the study: two electro spun thermoplastic nanofiber (Xantulayr^®^ from Revolution Fibres) with different areal weight, and one micro carbon fibers veil (Optiveil^®^ from TFP). Two laboratory made veils were also manufactured by electrospinning commercial polyethersulfone (PES) tougheners (Virantage by Solvay). The veils were selected to be either soluble or non-soluble in the epoxy resin matrix during curing. The solubility was analyzed by scanning electron microscopy and dynamic mechanical analysis testing on the cured laminates. The fracture energy was evaluated by double cantilever bending (DCB) testing under Mode I loading. The insoluble thermoplastic nanofibers showed the highest toughening efficiency, followed by the soluble nanofiber veils. The carbon fiber based veil showed no toughness improvement.

## 1. Introduction

Thermosets are widely used as a polymer matrix for fiber reinforced composites because of their easy processing and good mechanical properties. However, the inherent brittleness of thermosets led to the development of different toughening strategies [1,2]. Most of the approaches developed so far are optimized for prepreg technology only.

Among recent toughening approaches described in open literature, the use of electro spun veils is particularly interesting, because it may be used for both prepregging and resin infusion. Some authors have focused on non-dissolvable electro spun fibers based on the use of Nylon. Akangah et al. [3] showed that nylon-6,6nanofabric interleaving increased the threshold impact force by about 60%; the same nano fabric contributed to reducing the impact damage growth rate generated by drop-tower tests to one-half, and the related impact force from 0.115 to 0.105 mm^2^/N. Palazzetti et al. [4] confirmed similar results. Beckermann et al. [5] also reported a detailed study on nylon based veils commercialized by Revolution Fibers that are based on the same concept.

Some research teams have proposed an alternative approach based on the use of soluble veils which, after dissolving upon resin curing, undergo a phase separation that generates a specific phase morphology. Li et al. [6] showed that polysulfone veils can be dissolved in the interlaminar region, and therefore generate a particulate causing a 42% improvement in toughness compared to the baseline composites. Cicala et al. [7] showed that a full range of morphologies in the interlaminar region can be obtained by varying the veil content and the resin formulation. Magniez et al. [8] demonstrated that poly(vinylidene fluoride) (PVDF) electro spun veils can increase mode II toughness. The same authors also showed that it is possible to modify the thermomechanical properties depending on the PVDF veils by varying their molecular mass through a partial mix of the nanofibers. In a different paper, Magniez et al. [9] also demonstrated the use of Poly(Hydroxyether of Bisphenol A) nanofibrous veils as more efficient interlaminar tougheners compared to the partially mixed PVDF films because of their specific phase separation upon curing. 

Aside from being considered as toughening veils, soluble electro spun fibers can offer several other advantages. Their use to disperse carbon nanotubes in the interlaminar region upon veil dissolution was also reported [10], and Cicala et al. [11] extended this concept to develop multifunctional composites. Lionetto et al. [12] showed that this approach can be used to orient carbon nanotubes in a thermosetting matrix. Hamer et al. [13] showed that nylon electro spun fibers filled with carbon nanotubes were more efficient in terms of toughening than unfilled nylon electro spun fibers. POSS (polyhedral oligomeric silsesquioxanes) nanoparticles were selectively dispersed in the interlaminar region of composite laminates by using soluble electro spun nanofiber filled with the same type of nanoparticles [14]. Soluble fibers may, therefore, behave as nanoparticles carriers, a feature that insoluble fibers do not possess.

Currently, the only commercial toughening veil made of electro spun nanofibers is commercialized by Revolution Fibers under the tradename Xantulayr^®^. The use of the veil has increasingly gained interest because of its contribution to the increase of fracture toughness, compression after impact (CAI), and fatigue life in composites [15]. Xantulayr^®^ has received the AS9100c certification for use in the aerospace sector. These veils have been, however, characterized as prepregs inserts rather than on infusion-based systems. The toughening efficiency depends on a complex combination of matrix monomers [16,17] and cure profile [18]. The aim of this paper, therefore, is to compare veils made with insoluble fibers with the ones manufactured with soluble fibers when using the same resin system, the same reinforcement, and infusion processing techniques. The insoluble veils considered in this work include various types, from electro spun veils to carbon microfiber ones. The soluble veils were produced using laboratory-made polyethersulfone nanofibers starting from commercially available tougheners. 

## 2. Experiments

### 2.1. Materials and Methods

#### 2.1.1. Materials

The epoxy resin used in this work is diglycidyl ether of bisphenol A(DGEBA) supplied by Huntsman (Basel, Switzerland). The curing agent was 4,4′-methylene bis(2,6-diethylaniline) (MDEA) (Lonza, Basel, Switzerland). The thermoplastic polymers used for the soluble veils were two polyethersulfones (PES) purchased from Solvay (Bollate (MI), Italy,) and commercialized under the tradename Virantage (grades 10,200 and 30,500). The Virantage PES were developed as epoxy tougheners for prepreg with an average molecular weight of 46,500 g/mol and 23,000 g/mol for the 10,200 and 30,500 grades, respectively, as reported by Solvay. Two other types of veils were purchased from commercial sources. Nylon-based veils AP1500 and AP4500 of the Xantu.Layr series were purchased from Revolution Fibers (Henderson, Auckland, New Zealand). The carbon microfiber non-woven veils Optiveil were purchased from Technical Fibre Products Ltd. (Kendal, UK). Table 1 indicates the codes used in this paper to refer to the interlaminar veils considered.

Plain carbon fabrics (C-200 T from Prochima, PU, Italy) with an aerial weight of 200 gsm (grams per square meter) were used for the preparation of the reinforced samples. The unmodified epoxy matrix was prepared by mixing in stoichiometric amounts the hardener and the epoxy monomer at 80 °C for 30 min.

#### 2.1.2. Preparation of the Soluble Electro Spun Veils

The Virantage veils were prepared by dissolving 5.00 g of the PES powder in a solvent mixture (5.00 mL *N*,*N*-dimethylformamide [DMF] and 5.00 mL of Toluene) and stirring for 2 h at 40 °C. This solution was placed in a 3 mL medical syringe. PES veils were electro spun at a flow rate of 60 µL/min, 21 kV ddp and a 10 cm needle–collector gap onto a rotating drum (200 rpm) covered with the dried carbon fabric. Electrospinning was carried out on the NANON-01A equipment by MEC CO Ltd., Fukuoka, Japan.

#### 2.1.3. Composites Manufacturing

Six layers of dry carbon fabrics with veils laid up in the interlaminar region were stacked on a steel plate. An adhesive silicone tape was placed around the perimeter of the layered stack to provide a proper seal, and a flexible vacuum bag was placed on top. An inlet tube and an outlet tube were placed inside the vacuum bag. The inlet tube was connected by a valve to a pot filled with unmodified epoxy matrix, while the outlet tube was connected to a vacuum pump. The vacuum was applied when the inlet valve was closed to compact the layers and to remove excess air. All the stacked layers were placed in an oven preheated to 130 °C. The epoxy resin was vacuum infused into the stacked layers, which was maintained at 130 °C under a constant vacuum (−75 cm Hg). The temperature was kept at 130 °C for 30 min and then increased to 180 °C with a rate of 2 °C/min. The final temperature was maintained for 3 h. The required amount of PES veil was placed through the laminates according to the areal density of the carbon fabric (i.e., 200 gsm), to obtain 10 wt% or 20 wt% of the modifier agent in the final laminate. For the other non-soluble veils, only one layer was placed in the interlaminar region.

### 2.2. Characterization

#### 2.2.1. Dynamic Mechanical Analysis (DMA) of the Cured Composites

The viscoelastic behavior of the cured laminates was investigated using a TRITEC DMA analyzer (Triton Technology, Coventry, UK). The tests were carried out in single cantilever mode and samples of size (20 × 10 × 3) mm. The tests were performed at 1 Hz with a 2 °C/min heating rate ranging from 25 °C to 270 °C.

The electro spun veils were tested using the pocket DMA approach because they were too thin and brittle to be tested in tension or bending mode. The pocket DMA is a technique used in the pharmaceutical field [19] and for testing powders made of polymer blends [20,21]. Similar techniques have also been reported by Carlier et al. [21] to analyze the viscoelastic behavior of organic polymers. The test was carried out according to the following protocol. The veil was weighted (0.35 g) in a standard stainless steel pocket and pressed to obtain a uniform thickness. The pocket, filled with the pressed veil, was stabilized at 25 °C and then heated to 250 °C at 5 °C/min. Samples were cooled naturally and heated to 260 °C at 5 °C/min after the first scan. The results of the second are the ones illustrated in this paper. This technique allows for the direct evaluation of the thermal transitions from E’ (storage modulus) and tan δ. However, with the supported DMA, the absolute values of E’ and tan δ for the polymer are influenced by the presence of the metal pocket; the effective viscoelastic properties should therefore be analyzed by considering the sample as a sandwich structure. The tan δ versus temperature data have been reported in the paper.

#### 2.2.2. Scanning Electron Microscopy (SEM)

By using SEM EVO (Zeiss, Cambridge, UK) the morphology of the samples was investigated. The SEM analysis has been carried out on the electro spun veils and on the cured laminates. The electro spun veils were gold sputtered before the analysis without any other pre-treatment being applied. The diameter of the nanofibers was determined from the SEM images using the ImageJ software. The interlaminar regions of the composite laminates have been examined by SEM after polishing. A mixture of sulfuric acid/distilled water (3:2) was used for all the cured specimens to etch the epoxy and increase the contrast between the thermoplastic and epoxy phases [22]. For the etching treatment, the samples were immersed in the acid mixture and stirred for 20 min. After etching, the samples were washed with water and then sputtered with gold. The etching did not alter the phase morphology [16]. The fracture surface of the double cantilever bending (DCB) samples was also analyzed by SEM after acid etching.

#### 2.2.3. Double Cantilever Bending (DCB) Testing

Tests to evaluate the fracture toughness G_IC_ were carried out according to the ASTM5528 protocol for Mode I IFT for unidirectional fiber-reinforced polymer matrix composites. The reason behind the choice of this protocol relies on the fact that no specific standard is available to evaluate G_IC_ in woven fabric composites. The specimens were loaded by tensile wedge grips that held the hinges from both sides. Mode I loading was applied perpendicular to the crack plane at a constant crosshead speed of 1 mm/min by using an INSTRON 5988 (Instron, Milan, Italy) universal testing machine. One edge of each specimen was painted with white lacquer to assist the crack length measurements using a camera. Modified beam theory was used to calculate the G_IC_ values using the following equation:

(1) $$G_{IC}=\frac{3P\delta }{2B(a+\left|\Delta \right|)}.$$

In Equation (1) δ is the load point deflection (mm), *a* the delamination length (mm), and |∆| is a correction factor evaluated experimentally by plotting the cube root of the compliance C^1/3^ as a function of the delamination length. This correction factor compensates for shear deformation or displacements and rotations of the delamination front. The Mode I fracture energy measurements in this work refers to the “G_IC_ propagation’’ term in the technical nomenclature. Five samples have been tested for each type of laminate. The differences in mechanical properties arising from the tests have been statistically analyzed by using a one-way analysis of variance (ANOVA) with the Minitab 17 software platform. The comparison of the mean values was done using Fisher’s test with a 95% confidence level to identify which groups were significantly different from others.

## 3. Results and discussion

### 3.1. Veil Morphology

**Figure 1:**
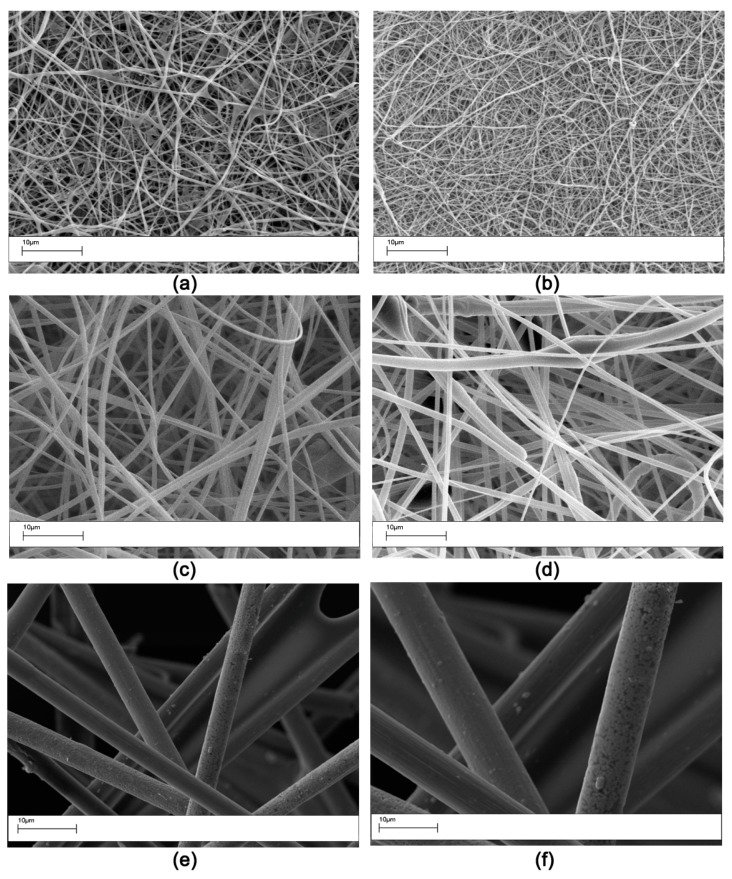
SEM morphology (Magnification 2000×) of the veils used: (**a**) AP1; (**b**) AP2; (**c**) V1; (**d**) V2; (**e**) OV; (**f**) OV with higher Magnification 5000×.

All the veils, with the exception of the OV one, displayed nanosized fibers (Figure 1, Table 2). The carbon fiber OV veil is made of micron-sized carbon fibers (≈7 µm) (Figure 1e). The carbon fibers showed a uniform particle coating (Figure 1f) with some resin strands spanning from one fiber to another. The resin residues are presumably the result of the application of a binder resin to hold the carbon fibers together.

### 3.2. Interlaminar Morphology for the Modified Composites

**Figure 2:**
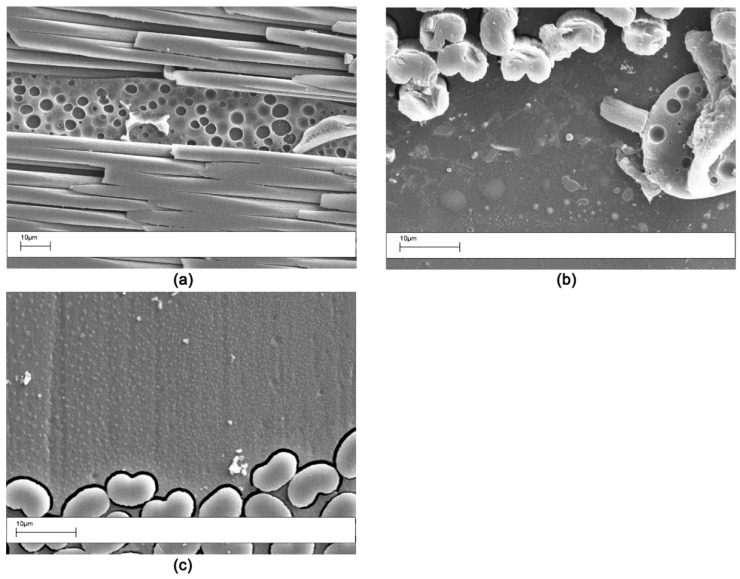
SEM morphology of the interlaminar region for the laminates modified with the soluble Virantage veils: (**a**) V1 (1000×); (**b**) V1 (2000×); (**c**) V2 (2000×).

The cured laminates were sectioned and the interlaminar morphology analyzed by SEM. The sample toughened by the V1 veil showed the coexistence of different phases, ranging from phase inversion to particulate. These phases were formed by the dissolution and separation of the veils in the curing resin (Figure 2). Only the V2 veil showed a fine particulate morphology (Figure 2c). This different behavior was the result of the different molecular mass in the two veils. Similar results were found in a previous paper focused on the use of PES veils [7]. The high molecular mass PES showed the occurrence of non-uniform phase morphologies.

**Figure 3:**
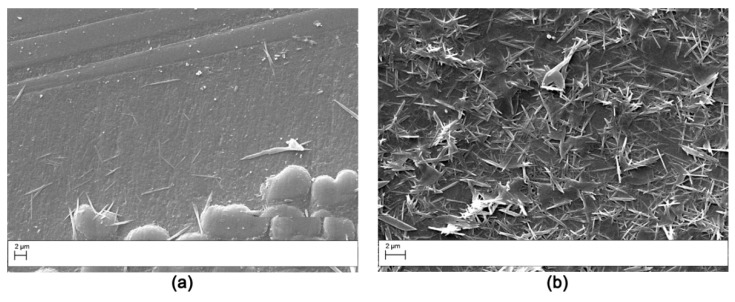
SEM morphology of the interlaminar region for the laminates modified with the insoluble veil AP1: (**a**) low magnification (4000×); (**b**) high magnification (8000×).

APs veils displayed no phase separation, and the nanofibers were clearly still visible in the interlaminar region after the curing of the resin (Figure 3). Similar results were previously reported [5,23]. The carbon fiber based OV veil also displayed a behavior already observed in open literature [24].

### 3.3. DMA Analysis for the Modified Composites

**Figure 4:**
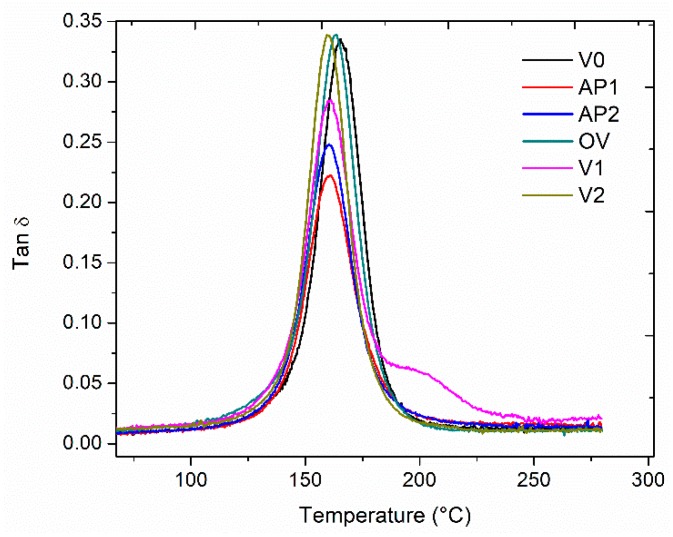
Tanδ versus temperature for the modified composites.

The viscoelastic properties of the laminates with veils uniformly placed through the thickness were evaluated by DMA testing. The unmodified laminate (V0) showed a clear tanδ peak centered at 165 °C due to the glass transition temperature of the epoxy resin (Figure 4). The samples modified with the veils AP1 and AP2 showed slightly lower glass transition temperatures at 162 °C and 160 °C, respectively. The tanδ peak height for these systems was lower compared to the one of the unmodified system. The insoluble micro carbon OV showed a negligible shift of the glass transition temperature to 164 °C. However, the peak height was unchanged. Similar results were observed for the laminate modified with 10 wt% of the V2 veil, but, in this case, the glass transition shifted down to 159 °C. The sample modified with 10 wt% of V1 veil showed a main tanδ peak centered at 160 °C but a shoulder appeared at 202 °C. The main tanδ peak height was lower than the unmodified system for the laminates containing the V1 veil.

**Figure 5:**
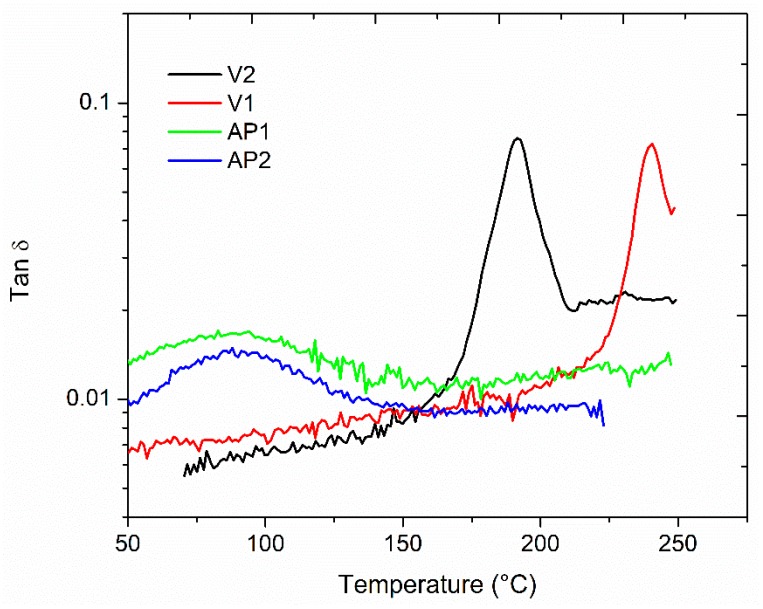
Tanδ versus temperature for the thermoplastic veils analyzed using supported dynamic mechanical analysis (DMA).

The veils adopted here were obtained using polymers showing different glass transitions and different solubility in the epoxy matrix. The insoluble APs veils showed Tgs centered at 85 °C, while the PES-based veils featured T_g_s at 240 °C and 191 °C for V1 and V2, respectively (Figure 5). The lower T_g_ of the V2 veil being explained by the lower molecular mass of V2 compared to V1. The lower Tgs of the APs veils can explain the slightly lower Tg measured for the laminates modified with these veils. The Tg reduction is not larger than few degrees only because the AP veils did not dissolve in the epoxy matrix, thus limiting their effect on the glass transition.

The presence of the AP veils resulted in the lowering of the tanδ peak height. The resulting peak observed is, therefore, the result of the plasticizing effect of the AP veils in each interlaminar region of the laminate. When micro carbon based veils OV were used, no tanδ peak height reduction was measured. Only a slight shift of the Tg was observed, probably due to the presence of the binder in the veil causing a minor plasticizing effect. In contrast, when the PES-based veils were used, the behavior varied according to the morphology of the system. The V1 modified laminate showed a shoulder at high temperature as the result of the presence of the phase inverted domains. However, the shoulder was at a lower temperature (i.e., 202 °C) compared to the Tg of the V1 veil (i.e., 240 °C). Similar results were found previously for similar systems [7]. The system modified by the V2 veils displayed a very fine particulate morphology (lower than 1 µm) that did not result in a clear separated tanδ peak, or in a shoulder in the tanδ curve.

### 3.4. DCB Properties of the Laminates

**Figure 6:**
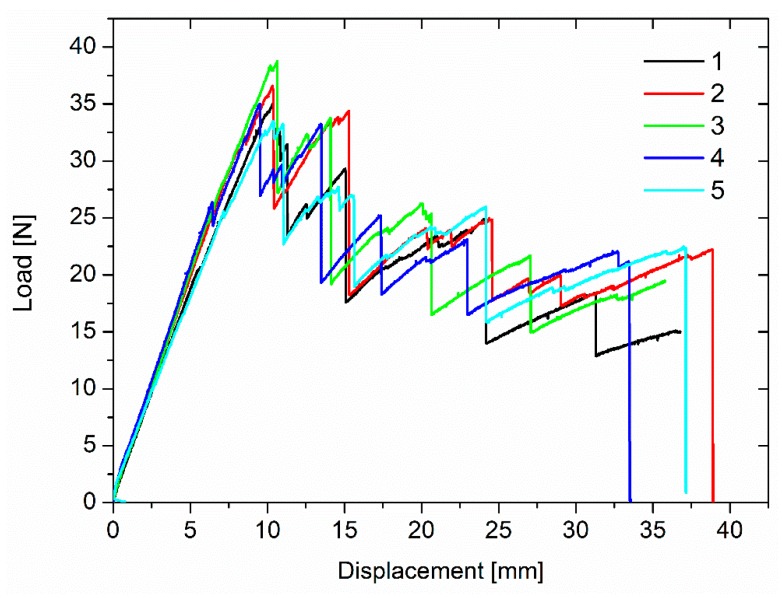
Typical load vs. displacement for the unmodified laminates.

Typical plots of the applied loads vs. deflections of the DCB specimens for the studied laminates are shown in Figure 6 for the unmodified laminates. The curves follow a stick–slip behavior common to composite laminates based on an epoxy matrix and woven fabric [24]. The unmodified specimens essentially exhibited a linear load increase up to the crack initiation; the crack propagation involved some significant jumps and was unstable. This behavior is typical of brittle samples, and a value of G_IC_ of 544.24 J/m^2^ was measured for this laminate.

**Figure 7:**
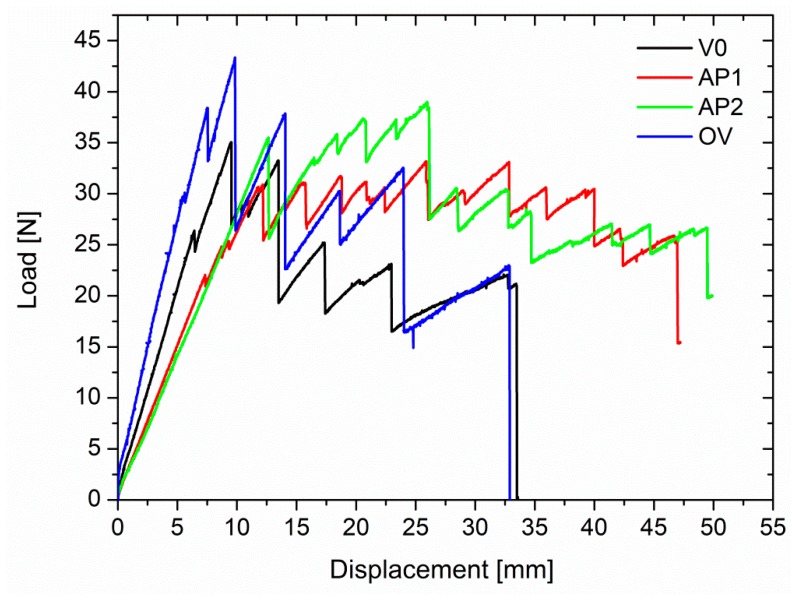
Typical load vs. deflection for the laminates modified with the insoluble veils.

**Figure 8:**
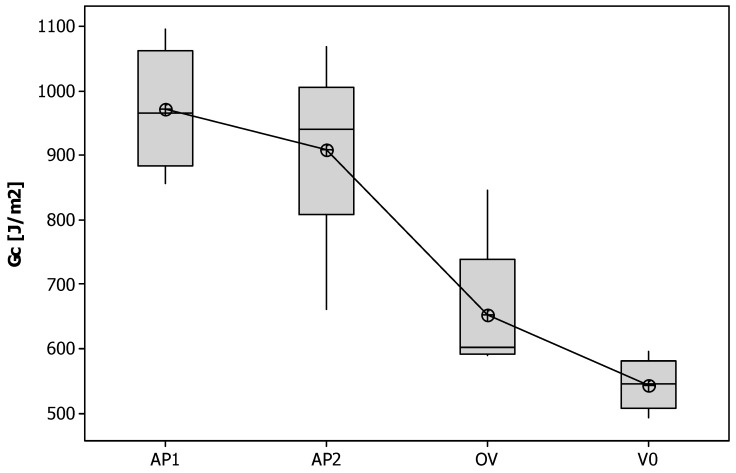
G_IC_ values for the laminates modified with insoluble veils.

The laminates modified with the insoluble veil showed similar behavior with nonlinearity and irregularities in the load increase as for the unmodified system (Figure 7). However, these samples showed multiple crack initiations, arrest mechanisms, and moderate load drop and increase cycles. This was evident in particular for the AP1 samples, and most likely due to the nanofiber bridging effect. The different behavior resulted in different G_IC_ values (Figure 8). The laminates reinforced with the OV veil did not displayed a G_IC_ (655.77 J/m^2^) significantly different from the one of the unmodified laminates (*p* = 0.071). AP1 and AP2 showed G_IC_ values of 971.23 J/m^2^ and 908.57 J/m^2^ significantly higher than the V0 sample (*p* = 0.000). AP1 and AP2 showed similar G_IC_ values (*p* = 0.413). The G_IC_ increased over the unmodified system of about 78% and 67% for AP1 and AP2, respectively.

**Figure 9:**
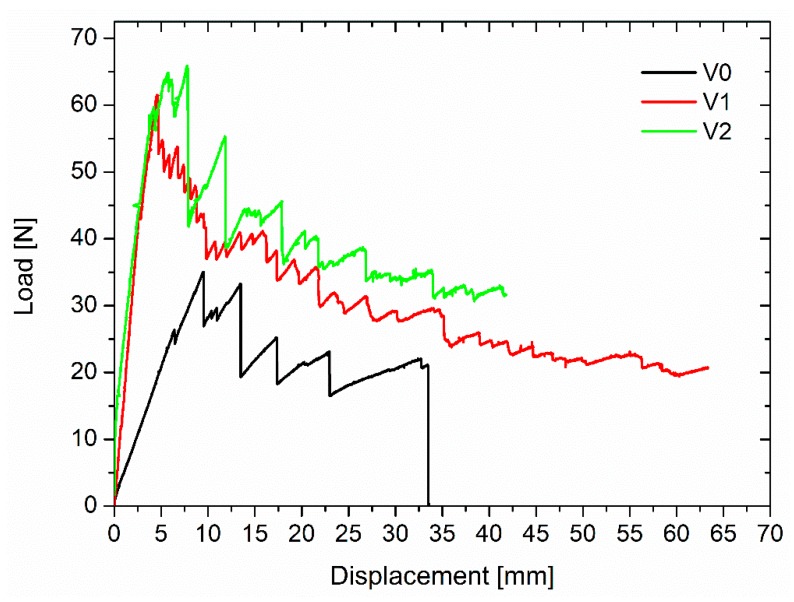
Typical load vs. deflection for the laminates modified with the soluble veils.

**Figure 10:**
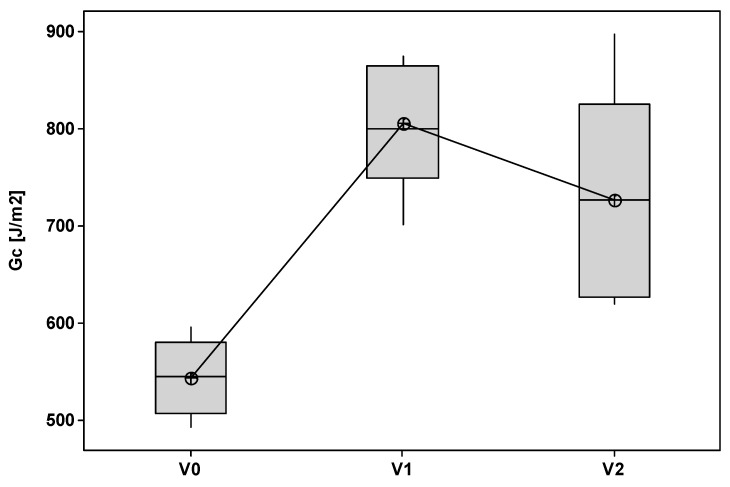
G_IC_ values for the laminates modified with soluble veils.

The PES soluble veils led to higher peak loads for crack initiation and smaller load drops during crack propagation compared to the unmodified systems (Figure 9). This difference in behavior with the systems modified with non-soluble veils was due to uniform phase separation observed in these systems. The presence of a uniformly dispersed thermoplastic rich domains can affect the crack propagation mode. The G_IC_ energy increased significantly (*p* < 0.001) from 544.44 J/m^2^ to 805.96 J/m^2^ and 726.61 J/m^2^ with increases, compared to the unmodified laminate, of 48% and 34% for V1 and V2 (Figure 10).

### 3.5. Fracture Surface after Mode I Testing

**Figure 11:**
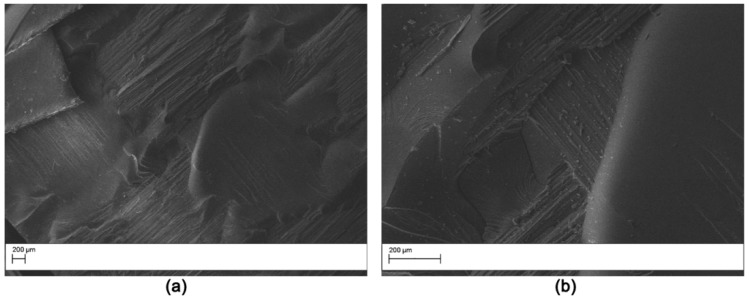
SEM of the fracture surface for the V0 double cantilever bending (DCB) sample: low magnification (50×) (**a**); high magnification (200×) (**b**).

**Figure 12:**
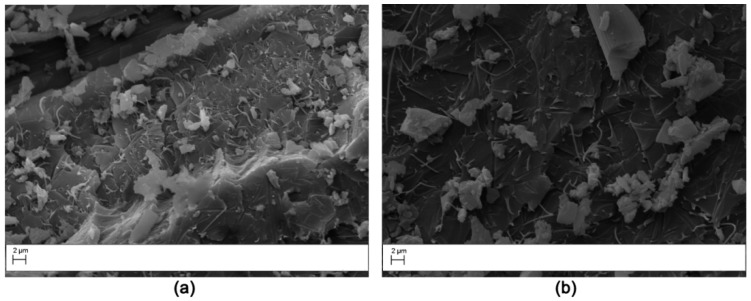
SEM of the fracture surface for the AP1 modified DCB sample (Magnification 5000×). The analysis was carried out on the two halves resulting by specimen opening. (**a**) right side, (**b**) left side.

**Figure 13:**
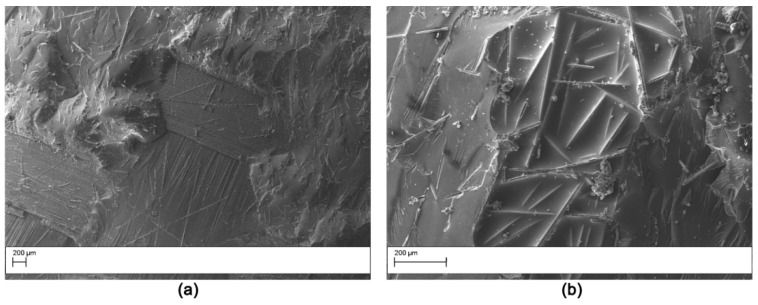
SEM of the fracture surface for the OV modified DCB sample: low magnification (50×) (**a**); high magnification (200×) (**b**).

The fracture surface of the DCB specimens after testing was evaluated by SEM analysis. The fracture surface of the V0 laminate (Figure 11) was smooth, indicating low crack growth resistance of the matrix due to the uninterrupted crack propagation in the continuous epoxy interlaminar layer. The samples modified with AP1 showed nanofibers pullout on both the two surfaces obtained from the DCB sample (Figure 12) indicating a cohesive failure mode. Similar results were observed for the AP2 sample. Hamer et al. [13] reported similar morphological features in epoxy laminates reinforced with Nylon 66 electro spun veils. Thermoplastic bridging is recognized as an efficient toughening mechanism for thermoset/thermoplastic blends when thermoplastic particles are used [25]. This toughening mechanism relies on the closure traction to the crack surfaces, and this leads to effectively reducing the local stress intensity factor at the crack tip. The nanofibers bridged the two crack separating surfaces showing that insoluble thermoplastic nanofibers can effectively induce thermoplastic bridging. The OV modified laminates showed a brittle surface with micro carbon fiber well embedded in the matrix (Figure 13). No bridging effect was displayed for this sample.

**Figure 14:**
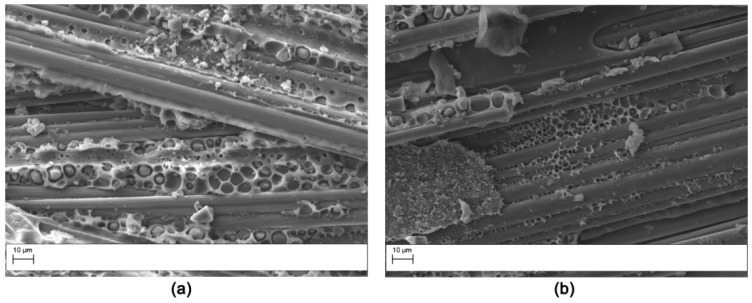
SEM of the fracture surface for the V1 modified DCB sample (Magnification 2000×). The analysis was carried out on the two halves resulting by specimen opening. (**a**) right side, (**b**) left side.

**Figure 15:**
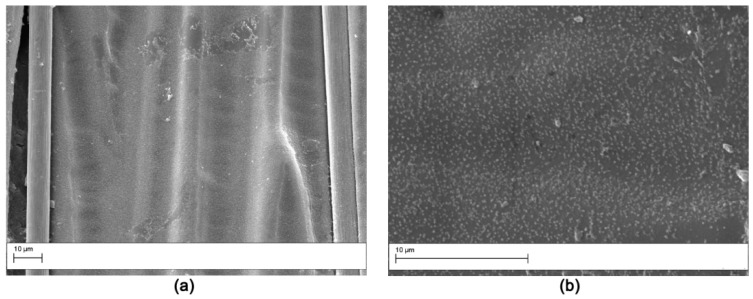
SEM of the fracture surface for the V2 modified DCB sample: low magnification (2000×) (**a**); high magnification (10,000×) (**b**).

The laminates modified with PES veils displayed a unique fracture surface due to the polymer phase separation. The V1 sample showed extensive phase inversion and resin hackles on the carbon fibers were observed (Figure 14). The V2 sample showed similar features, but the phase morphology was finely particulate (Figure 15) as already observed in the cross-section analysis (Figure 3). The nodular structure of the phase inverted domains (Figure 14) showed a ductile failure leading to a toughening effect by the absorption of the fracture energy due to the presence of the thermoplastic-rich phase. The fracture surface for the V2 sample appeared more uniform and smoother than in the case of the V1 sample; this was consistent with smaller toughening increases measured for the laminates reinforced with this veil.

## 4. Conclusions

Different veils were analyzed in this paper. The use of non-soluble veils proved to be an efficient way to toughen the laminates only when nanosized thermoplastic fibers were used. This result was mostly due to the bridging effect provided by these fibers. From this point of view, the development of tailored surface chemistry for these veils for enhanced fiber/matrix adhesion could be beneficial to further increase the toughening efficiency. However, the use of a thermoplastic matrix with lower Tg (≈85 °C) compared to the neat epoxy matrix resulted in a slight decrease (i.e., ≈3–5 °C) of the glass transition temperature, and also led to a significant change in the tanδ values. This limitation could be overcome by selecting thermoplastic grades with slightly higher Tgs, or by tailoring the veil areal weight to reduce the veil/resin ratio.

The veil based on micro carbon fibers did not provide a significant effect on the viscoelastic behavior. The carbon fibers were not sufficiently efficient to toughen the laminates because no strong energy dissipation mechanism was activated by these veils.

The soluble veils showed mechanical properties depending on the molecular mass of the polymer. The veil with the lowest molar mass (i.e., V2) showed the smallest toughening improvement compared to high molar mass veil. This result largely depended on the phase morphology formed. It must, however, be observed that the study was limited to a certain amount of PES veil (i.e., 10 wt%), and this might change with the use of higher veil contents.

These results offer an improved knowledge about the behavior of different interlaminar toughening veils. Further research should be carried out to extend the study to resins with higher functionalities, and by varying the morphologies generated by the soluble veils to draw some robust guidelines about the use of these veils.

## Figures and Tables

**Table 1:** Tested.

Sample Code	Interlaminar Modifier
**Base**	None
**V1**	Virantage 10,200
**V2**	Virantage 30,500
**AP1**	AP1500
**AP2**	AP4500
**OV**	Optiveil

**Table 2:** Properties.

Veil	Diameter Avg [nm]	Areal Weight [g/m^2^]
**V1**	873	1.50
**V2**	450	4.50
**AP1**	165	1.68
**AP2**	348	2.48
**OV**	7864	11.72
